# Cognitive deficits in adults with obstructive sleep apnea compared to children and adolescents

**DOI:** 10.1007/s00702-015-1501-6

**Published:** 2016-01-04

**Authors:** Krzysztof Krysta, Agnieszka Bratek, Karolina Zawada, Radosław Stepańczak

**Affiliations:** 1 Department of Psychiatry and Psychotherapy, Medical University of Silesia, ul. Ziołowa 45/47, 60-635 Katowice, Poland; 2 Department of Pneumonology, Medical University of Silesia, ul. Medyków 14, 40-752 Katowice, Poland; 3 Mental Health Center FENIKS, ul. Grunwaldzka 46, 41-800 Zabrze, Poland

**Keywords:** Sleep apnea syndromes, Cognitive science, Obstructive sleep apnea, Neuroimaging, Neuropsychological tests

## Abstract

Obstructive sleep apnea (OSA) can negatively affect the patient’s physical and psychological functioning, as well as their quality of life. A major consequence of OSA is impaired cognitive functioning. Indeed, several studies have shown that OSA mainly leads to deficits in executive functions, attention, and memory. As OSA can present in all age groups, these associated cognitive deficits have been observed in adults, as well as in children and adolescents. However, these cognitive deficits may have a different clinical picture in young patients compared to adults. In this review, we analyze the most affected cognitive domains in adults and children/adolescents with OSA, as evaluated by neuropsychological and neuroimaging studies. We found that deficits in working memory, attention, or executive functions cognitive domains are found in both adults and children with OSA. However, children with OSA also show changes in behavior and phonological processing necessary for proper development. Moreover, we examine the possible OSA treatments in children and adults that can have a positive influence on cognition, and therefore, improve patients’ general functioning and quality of life.

## Introduction

While physiological changes in breathing normally occur during the different sleep phases, more profound breathing alterations are observed in some pathological sleep disorders, such as in obstructive sleep apnea (OSA) (Atanasov and Dimov 2003). OSA is characterized by repeated episodes of closure or stenosis of the upper respiratory tract, leading to apnea or hypopnea with either preserved normal or increased respiratory muscle activity. In adults, the clinical history of OSA includes nocturnal symptoms such as periods of apnea, interrupted by periods of loud snoring, restless sleep, and sudden awakenings. Additionally, daytime symptoms such as increased sleepiness, morning headaches, and impaired attention have been observed (Guilleminault et al. 1977). Obesity is the most common cause of OSA in adults (Chen et al. 2014), while other factors, such as high neck circumference, craniofacial and upper airway anatomical abnormalities, can also increase the likelihood of developing OSA (Borges et al. 2014). OSA requires differentiation with Obesity Hypoventilation Syndrome (OHS) which concerns obese patients, presenting clinical signs of OSA but not confirmed in polysomnography (Zawada et al. 2013; Ryan et al. 2014; Liu and Yuan 2014).

The clinical picture of OSA in children or adolescents is different to adults. Children with OSA present with loud snoring, together with other, non-specific symptoms, including: growth retardation, enuresis, academic learning difficulties, and behavioral problems, such as attention deficit/hyperactivity (ADHD) disorders. Unlike obesity in adults, tonsillar and adenoid hypertrophy is the most common etiology of OSA in children, followed by anatomical abnormalities (Solyom et al. 2014). Despite the different clinical presentations, polysomnography remains the gold standard for diagnosing OSA in adults and children, in addition to clinical history (Health Quality Ontario 2006).

As a consequence of OSA, decreased blood oxygen saturation, and conscious or unconscious arousal (wake-up events) can occur during the night (Paiva and Altarian 2014). If left untreated, OSA may cause significant neurological problems, including stroke, depression, headaches, peripheral neuropathy, and non-arteritic ischemic optic neuropathy (NAION). Moreover, OSA is often associated with impaired cognitive function, most likely due to the intermittent hypoxia.

A relationship between OSA and cognitive impairment was first identified in the 1980s (Findley et al. 1986; Greenberg et al. 1987; Klonoff et al. 1987). However, despite many years of research in this field, the prevalence, pathogenesis, and therapy of impaired cognitive functions relating to OSA remains elusive. Moreover, in comparison to a wide range of publications devoted to cognition in the adult population with OSA, research on the neuropsychological effects of the pediatric form of the disorder is limited (Beebe et al. 2004; Horiuchi et al. 2014).

In this review, we aimed to determine the most affected cognitive domains in adults and children/adolescents with OSA, as evaluated by neuropsychological and neuroimaging studies. To do this, we identified previously published studies on sleep, breathing disorders, and cognitive dysfunction from 1990 until 2015 the PubMed database using the following search terms [effective date: August 26, 2015): (sleep (Title/Abstract) AND apnea (Title/Abstract)] OR breathing disorders (Title/Abstract) OR cognitive deficits (Title/Abstract) OR attention (Title/Abstract) OR executive functions AND cognitive deficits (Title/Abstract)] OR children (Title/Abstract) OR adolescents OR adults OR CPAP OR polysomnography AND English (lang) AND (1990/01/01(PDAT): 2015/08/26PDAT)). We identified 26 original studies referring to adults, and 15 original studies referring to children and adolescents. We also included systematic reviews, meta-analyses, case reports, etc., in our discussion.

### Cognitive domains affected in adults with OSA

Overviews of the identified studies investigating cognitive deficits due to OSA in the adult population are presented in Tables 1 and 2. Unfortunately, the exact prevalence of the cognitive dysfunctions in adults with OSA is still unknown and varies widely depending on study design. For example, in one study (Saunamäki et al. 2009), most OSA patients performed at a normal level; only few patients had mild (2.5–12.5 %) or moderate-to-severe (5–15 %) cognitive dysfunction. In contrast, a prospective observational study reported that one in four newly diagnosed OSA patients had a severe and distinctive neuropsychological dysfunction (Antonelli Incalzi et al. 2004). Finally, Pierobon et al. (2008) reported that at least 59.2 % of obese OSA patients had at least one cognitive impairment. Therefore a large, multi-center study on the cognitive deficits in OSA patients, with and without obesity, is still required to accurately determine its prevalence.

**Table 1:** Neuropsychological assessment of cognitive impairments in adults with obstructive sleep apnea syndrome—an overview of selected studies published after year 2000

Study	Study group	Methods	Results
Rouleau et al. (2002)	28 OSA patients 18 controls	Mirror tracing; rotary pursuit skill learning tasks	No significant differences in procedural skill learning abilities in OSA patients compared to controls
Sforza et al. (2004)	152 OSA patients 45 controls	PVT	Lower speed and accuracy in OSA patients
Verstraeten et al. (2004)	36 OSA patients 32 controls	TMT; SDMT; digit span forward and backward; Stroop color-word test; five-point design fluency; Attentional flexibility task	Lower performance of OSA patients in the SDMT, digit span forward task, and attentional flexibility task
Mazza et al. (2005)	20 OSA patients 40 controls	OSLER, CPT, and driving simulator tests at three time sessions	Worse performance in all three attentional tests at the three time sessions in OSA patients
Naëgelé et al. (2006)	95 OSA patients 95 controls	Extensive battery of tests evaluating verbal episodic, procedural, and working memory	Overall lower performance in all tests in OSA patients
Quan et al. (2006)	67 mild to moderate OSA patients 74 controls	Wechsler Adult Intelligence Test-Third Edition; Stroop test; TMT; Grooved pegboard test	No significant differences in any test in OSA patients compared to controls
Lis et al. (2008)	20 OSA patients 10 controls	N-back test	Deficits in *n*-back tasks in OSA patients regarding accuracy and reaction times
Pierobon et al. (2008)	157 obese OSA patients	Digit symbol test; TMT	59.2 % of OSA patients were impaired in at least one cognitive function
Saunamäki et al. (2009)	40 newly diagnosed male OSA patients 20 controls	RCFT; block design, TMT; intra-extra dimensional set shifting test	Generally lower performance in all tests in OSA patients
Yaouhi et al. (2009)	16 newly diagnosed OSA patients 14 controls	TAP; Wechsler memory scale (WMS3); verbal fluency test; Purdue pegboard test	Worse performance in two of the WMS3 episodic memory subtests and Purdue pegboard test in OSA patients
Bawden et al. (2011)	17 OSA patients 20 controls	MMSE; brief cognitive screening battery; digit-symbol test; phonemic verbal fluency	Significantly worse performance in all administered tests in OSA patients
Canessa et al. (2011)	17 treatment-naive OSA patients 15 controls	Raven; digit-span test; Corsi task; Rey-list; Stroop test; TMT; paced auditory serial addition test	Overall lower performance in all tests in OSA patients
Nemeth et al. (2012)	20 OSA patients 20 controls	Listening span task; alternating serial reaction time task	Preserved general skill and sequence-specific learning, and impaired working memory performance in OSA patients
Torelli et al. (2011)	16 OSA patients 14 controls	Rey auditory-verbal learning test; Stroop test; digit-span test	Lower performance in Rey auditory-verbal learning test, Stroop test and digit span backward scores in OSA patients
Borges et al. (2013)	22 OSA patients without comorbidities 22 controls	Test battery that included measures of six distinct executive domains	No significant group differences in all test scores in OSA patients compared to controls
Joo et al. (2013)	38 severe male OSA patients 36 controls	Korean California verbal test; RCFT; digit-span test; Corsi block-tapping tests; Korean Boston naming test	Significantly decreased speed of processing, verbal and visual attention, and memory in OSA patients
Tulek et al. (2013)	24 OSA patients 14 controls	Simon, Flanker and Stroop tasks	Deficient attentional control processes when focal attention (Flanker task) processes were involved
Sales et al. (2013)	14 OSA patients 13 controls	Toulouse-Pieron attention test; WCST; digit symbol substitution test; digit-span test; similarities test; logical memory and verbal paired association tests; RCFT	Worse performance in attention tests and tests of long-term memory and working memory/executive function in OSA patients
Djonlagic et al. (2014)	20 newly diagnosed OSA patients 20 controls	PVT; MST in the evening and in the morning	Similar learning in the evening, but significantly less overnight improvement on the MST in OSA patients

*CPT* continuous performance task, *MST* motor sequence learning task, *OSA* obstructive sleep apnea, *OSLER* oxford sleep resistance, *PVT* psychomotor vigilance task, *RCFT* Rey complex figure test, *SDMT* symbol digit modalities, *TAP* test battery for attentional performance, *TMT* trail-making test, *WCST* Wisconsin card sorting test

**Table 2:** Neuroimaging assessment of adults with obstructive sleep apnea—an overview of selected studies published after year 2000

Study	Study group	Methods	Results
Morrell et al. (2003)	7 right handed male, newly diagnosed OSA patients 7 controls	Voxel-based morphometry	Significantly lower gray matter concentration within the left hippocampus in OSA patients but no difference in total gray matter volume
Alchanatis et al. (2004)	22 severe OSA patients 10 controls	Proton MRS	Lower *N*-acetylaspartate-to-creatine ratio, choline-to-creatine ratio, and absolute concentrations of N-acetylaspartate/choline in the frontal white matter in OSA patients
Bartlett et al. (2004)	8 males with OSA 5 controls	MRS of hippocampus; PVT; digit symbol substitution task	Significantly increased *N*-acetyl-containing/creatine-containing compounds in the left hippocampal area in OSA patients Lower levels of hippocampal creatine-containing compounds were correlated with a worse neurocognitive performance
Thomas et al. (2005)	16 OSA patients 16 controls	fMRI; 2-back verbal working memory task	Significantly slower working memory speed and absence of dorsolateral prefrontal activation in OSA patients
Macey et al. (2008)	41 OSA patients 69 controls	Diffusion tensor imaging	Lower fiber integrity in multiple regions
Ayalon et al. (2009)	14 OSA patients 14 controls	fMRI; Go/no-go task	Lower ability to withhold a response in OSA associated with decreased brain activation
Yaouhi et al. (2009)	16 newly diagnosed OSA patients 14 controls	MRI; (18)FDG-PET	Gray matter loss in the frontal and temporo-parieto-occipital cortices, the thalamus, hippocampal region, some basal ganglia and cerebellar regions in OSA patients Hypometabolism in the right hemisphere, precuneus, middle and posterior cingulate gyrus, parietal cortex, occipital cortex and superior temporal gyrus in OSA patients
Canessa et al. (2011)	17 treatment-naive OSA patients 15 controls	MRI	Focal reductions of gray-matter volume in the left hippocampus, left posterior parietal cortex, and right superior frontal gyrus in OSA patients
Torelli et al. (2011)	16 OSA patients 14 controls	MRI	Lower volumes of cortical gray matter, right hippocampus, and caudate nuclei in OSA patients
Kumar et al. (2014)	43 newly-diagnosed OSA patients 61 controls	MRI	Putamen areas with increased and decreased tissue volumes in OSA patients

*(18)FDG-PET* 18-fluoro-deoxyglucose positron emission tomography, *fMRI* functional magnetic resonance imaging, *MRI* magnetic resonance imaging, *MRS* magnetic resonance spectroscopy, *OSA* obstructive sleep apnea, *PVT* psychomotor vigilance task

**Fig. 1:**
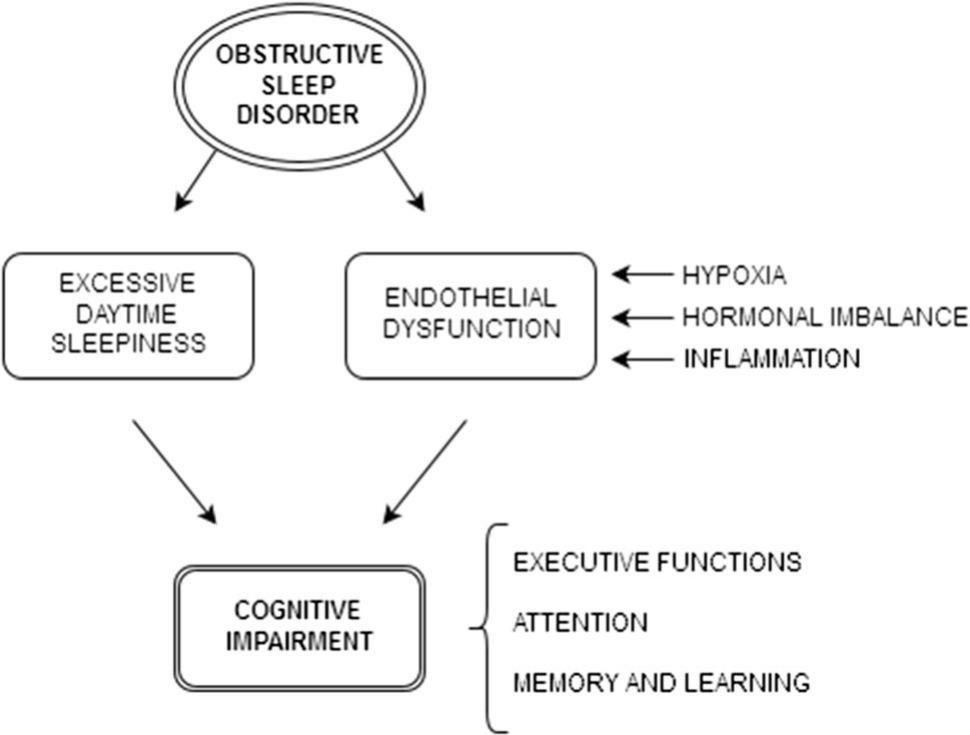
Impact of OSA on cognitive impairment

According to a recent meta-analysis (Bucks et al. 2013), most published studies have identified executive functions, attention, and memory as the most affected cognitive domains in OSA. Moreover, a systematic review published by Saunamäki and Jehkonen (2007) stated that executive functioning is the most impaired cognitive domain in OSA in the adult population (Fig. 1).

#### Executive function is impaired in adults with OSA

Executive function is the control of cognitive processes, including working memory, reasoning, task flexibility, problem solving, planning, and execution. Deficits in executive function in adults with OSA are mostly related to working memory, but also to phonological fluency, cognitive flexibility, and planning (Saunamäki et al. 2009). However, while the executive functions in the systematic review by Saunamäki and Jehkonen (2007) were generally assessed with standardized test methods, the sample sizes were significantly varied and the groups were widely distributed in terms of OSA severity. Nemeth et al. (2012) suggested that there is selective susceptibility of OSA patients to the more attention-demanding cognitive functions, such as working memory and executive functions, compared to general skill learning and sequence-specific learning. Moreover, when Lis et al. (2008) separated the deficits in executive functions into the more elementary cognitive processes involved in task solving, they found differences in processing time derived from the deficits at a more basic cognitive processing level. However, this reduced accuracy appeared to be attributed specifically to working memory.

On the other hand, one study (Borges et al. 2013) on a selected group of OSA patients without comorbidities reported no significant group differences in test scores measuring six distinct executive function domains (shifting, inhibition, updating, dual-task performance, planning, and access to long-term memory). The authors of this study concluded that OSA without comorbidities did not lead to executive function impairment. However, Hilsendager et al. (2015) reported that OSA combined with obesity does lead to executive function impairment, and that obesity status is a significant predictor of performance in the executive functioning tasks.

In summary, the previously published studies suggest that working memory is the most commonly affected subpart of executive functioning in adults. However, it remains to be established whether these changes are related to OSA symptoms or the comorbidities, such as obesity, which lead to cardiovascular and neurological complications. A valuable assessment of working memory using one consistent neuropsychological test, supported by neuroimaging studies discussed below, should be performed in order to confirm these findings.

#### Attention deficits are common in adults with OSA

Several studies have demonstrated that OSA patients show impairments in sustained, selective, and divided attention (Bucks et al. 2013; Gagnon et al. 2014). It has been suggested that these vigilance and attention deficits could influence and worsen other aspects of cognitive deficits in OSA, such as executive functioning and episodic memory impairments (Gagnon et al. 2014). However, most of these studies included only moderate and severe OSA patients, while mild OSA subjects were generally excluded.

Not all aspects of attention are equally impaired in OSA patients. In one study (Tulek et al. 2013), attentional control processes were found to be impaired when focal attention (Flanker task) processes were involved, but were intact when observed using the Simon and Stroop tasks. Moreover, Sforza et al. (2004) found that the speed with which the subjects respond to a visual stimulus (measured with the psychomotor vigilance task) was more affected than the accuracy in OSA patients.

As Mazza et al. (2005) hypothesized that a single test could underestimate impaired vigilance and attention in some patients, they performed three attentional tests (measuring maintenance of wakefulness, as well as sustained, selective, and divided attention) at three time points (9 am, 11 am, and 1:30 pm) within the same day. OSA patients performed significantly worse in all three tests during the three sessions. Additionally, vigilance and/or attentional impairments were found in 95 % of OSA patients. While the results of this study show a high percentage of OSA patients with attentional deficits, the results could have been influenced by morning somnolence, which is more pronounced in OSA patients. Therefore, it would be valuable to perform a battery of attentional deficits tests, at different times of the day and while performing different activities, in both mild and moderate/severe cases of OSA.

#### Memory and learning deficits are often mild in adults with OSA

Most studies report that memory impairment in OSA patients is mild (Yaouhi et al. 2009; Gelir et al. 2014), and not all memory processes are affected (Beebe et al. 2003; Naëgelé et al. 2006; Wallace and Bucks 2013). According to a recent meta-analysis (Wallace and Bucks 2013), subjects with OSA are more significantly affected in verbal episodic memory and visuo-spatial episodic memory, but not as much in visual immediate recall or visuo-spatial learning when compared to healthy controls. However, the authors emphasize the weaknesses in the methodology of the reviewed publications, such as significant heterogeneity in the sample size, oxygen levels, premorbid IQ or education, choice of comparison group, and a lack of classification for memory tasks. They also discuss the possible necessity to use screening polysomnography in the control group.

Twigg et al. (2010) reported that OSA is associated with an impairment in verbal, but not visual memory. This contrasts with the meta-analysis conducted by Beebe et al. (2003), who found no significant effect of OSA on verbal immediate recall and verbal long-term memory. Moreover, Naëgelé et al. (2006) investigated three separate memory systems (verbal episodic, procedural, and working) and found OSA patients exhibited a deficit of episodic memory, decreased performance in procedural memory, and an impairment of specific working memory capabilities. However, no correlation was found between OSA severity and memory deficits, which is in line with the results of Twigg et al. (2010). On the other hand, one group recently reported negative correlations between OSA severity using the apnea-hypopnea index (AHI) and immediate memory, logical memory, and theme drawings (Jurádo-Gámez et al. 2015). In contrast, Gelir et al. (2014) found no significant effect of OSA on working or procedural memory, which is in line with the study of Rouleau et al. (2002).

In summary, the data have been inconsistent in terms of impaired memory functioning in OSA patients. Such inconsistencies could be related to a difficulty in finding a proper diagnostic neuropsychological test that would precisely examine this type of dysfunction. It would be valuable to assess the cortical and cerebellar areas responsible for each type of memory system using a neuroimaging studies as a supplement to a valid neuropsychological test as discussed below.

### Cognitive deficits in adults with OSA assessed by neuroimaging studies

Neuroimaging assessment in conjunction with neuropsychological studies can help us to confirm the cognitive deficits in OSA. The studies that have used such neuroimaging techniques are summarized in Table 2.

Zhang et al. (2011) assessed severe OSA patients with both neuropsychological and neuroimaging tests (i.e., functional magnetic resonance imaging, fMRI, during a brief visual delayed matching-to-sample task), and showed a reduced mismatch-related activation in frontal regions. This impairment correlated with the arousal index and hypoxia in these patients, suggesting that hypoxia may cause this cognitive deficit. In a similar study (Thomas et al. 2005), fMRI was used to map cerebral activation during a 2-back verbal working memory task. Working memory speed in OSA patients was significantly slower than in the control group and was associated with the absence of dorsolateral prefrontal cortex activation. Therefore, by combining these neuropsychological and neuroimaging tests, it was confirmed that working memory is affected in adults with OSA.

As the hippocampus and frontal cortex are closely associated with memory processes and executive functions, and are particularly susceptible to sleep fragmentation, hypoxemia and hypercapnia (Beebe and Gozal 2002), most studies show altered brain metabolism and neuronal loss in these areas in OSA patients. For example, Alchanatis et al. (2004) examined OSA patients using proton magnetic resonance spectroscopy (MRS) and found that the *N*-acetylaspartate-to-creatine ratio (indicative of axonal loss and/or dysfunction) and the choline-to-creatine ratio (indicative of myelin lipid loss and or/phospholipid metabolism dysfunction) were significantly lower in the frontal white matter of OSA patients compared controls. In regards to the frontal lobe, white matter lesions are known to be associated with a cognitive executive dysfunction, which may explain the irreversibility of some cognitive deficits related to OSA. Moreover, a study performed on hippocampal area metabolites (Bartlett et al. 2004) reported that in the left hippocampal area, the *N*-acetyl-containing:creatine-containing compound ratio was significantly increased in OSA subjects. As there was no significant difference in the hippocampal *N*-acetyl-containing compounds levels, the observed alterations were most likely due to a decrease in creatine-containing compounds. These lower levels of hippocampal creatine-containing compounds were correlated with a worse OSA severity and neurocognitive performance. Finally, using voxel-based morphometry, Morrell et al. (2003) reported a significantly lower gray matter concentration within the left hippocampus in right-handed OSA patients compared to controls. The results of these studies indicate the brain morphology changes in the hippocampus are associated with OSA.

Similarly, Yaouhi et al. (2009) reported that significant cerebral changes, as assessed by MRI, precede the onset of neuropsychological impairment in patients with OSA. Indeed, indications of axonal injury in axons linking major structures within the limbic system, pons, frontal, temporal, and parietal cortices and projections to and from the cerebellum have been reported in OSA patients (Macey et al. 2008). Torelli et al. (2011) also evaluated brain morphological changes and their relationship to neuropsychological test results in moderate to severe OSA patients. The volumes of cortical gray matter, as well as the size of the right hippocampus, right and left caudate, were smaller in OSA patients compared to the control group and correlated with poorer performance in several neuropsychological tests. In addition, when brain activation during a response inhibition test (i.e., a Go/No-go task) was examined using fMRI, OSA patients showed lower ability to withhold a response, and this was associated with decreased brain activation in the left postcentral gyrus, cingulate gyrus, and inferior parietal lobe, right insula and putamen (Ayalon et al. 2009). Therefore, different areas in the brain can be morphologically affected in OSA patients, and these changes correspond to differing cognitive deficits.

By combining neuropsychological assessment and neuroimaging studies, we can precisely identify the areas in the brain responsible for cognitive impairments in OSA patients. There should be more focus on identifying the particular areas in the brain that are responsible during assessment of OSA patients with cognitive deficits. Such combined studies would help us to determine whether these changes are due to hypoxemic episodes of OSA or other causes.

### Cognitive deficits in children and adolescents with OSA

While there are only limited studies on cognitive deficits in children and adolescents, the accessible findings show that the type and intensity of symptoms presented in young patients vary according to age. Unfortunately, while there is substantial evidence confirming the presence of a cognitive deficit in school-aged children, the number of studies examining pre-school children is much lower (Jackman et al. 2012). A summary of the available studies on the cognitive deficits in children and adolescents with OSA is shown in Table 3.

**Table 3:** Neuropsychological and neuroimaging assessment of cognitive impairments in children and adolescents with obstructive sleep apnea syndrome—an overview of selected studies published after year 2000

Study	Study group	Methods	Results
Gottlieb et al. (2004)	205 SBD patients (aged 5 years)	NEPSY attention and executive core domain; NEPSY memory core domain; CPT called catch the cat; WPPSI-R	SDB symptoms associated with poorer executive function and memory skills and lower general intelligence
Archbold et al. (2004)	12 mild SDB patients (5 girls, 7 boys, aged 8.0–11.9 years)	CPT; CCT	Significant impairment of sustained attention and vigilance on a computerized CPT in SBD patients
O’Brien et al. (2004)	35 SDB patients (aged 6.7 ± 0.6 years)	Preschool form of the DAS; NEPSY	SDB patients had lower scores attention/executive function domain, specifically on visual attention and executive function. SBD patients had significantly lower scores on phonological processing
Hamasaki Uema et al. (2007)	81 OSA patients (aged 6–12 years)	RAVLT; WISC-III	Children with OSA show worse Rey test scores
Bourke et al. (2011)	59 PS patients (aged 7–12 years) 24 mild OSA patients 19 moderate/severe OSA patients	WASI; WRAT-3; RCFT; COWAT	Neurocognitive deficits common in children with PS, and mild/moderate/severe OSA
Biggs et al. (2011)	127 SDB, PS, mild/moderate/severe OSA patients (aged 7–12 years)	BRIEF; CogHealth	4 years after tonsillectomy, improvements in SDB concomitant with improvements in some areas of neurocognition, but not academic ability or behavior in school-aged children
Miano et al. (2011)	13 PS patients 31 OSA patients	ADHD rating scale; WRAT-3	Arousal as a defensive mechanism may preserve cognitive function by counteracting the respiratory events, at the expense of sleep maintenance and NREM sleep instability
Kheirandish-Gozal et al. (2011)	10 polysomnographically diagnosed OSA patients (aged 7–10 years)	Stroop color-word task; empathy task; fMRI scanner	Cognitive and empathetic processing is deteriorated in OSA children
Jackman et al. (2012)	60 PS patients (aged 3–5 years) 32 mild OSA patients 24 moderate/severe OSA patients	NEPSY; RBMT; DA; SR; ABAS-II	SDB of any severity is associated with poorer behavior but not cognitive performance
Quan et al. (2013)	43 SDB patients (aged 6–11 years)	SWMT; Simple reaction time; Multiplexing task	Long-term changes in executive function are not detectable with neurocognitive testing, but visible using neuroelectrophysiological monitoring
Tan et al. (2014)	31 OSA patients (aged 10–18 years, mean BMI = 32.3)	WASI; wide range assessment of memory and learning 2; WIAT-II; behavioral assessment system for children 2; behavior rating inventory of executive function	OSA increases the risk for some poorer educational and behavioral outcomes
Hannon et al. (2012)	37 adolescents with OSA (aged 12–18 years; BMI > 97th percentile)	WASI, RAVLT; list B recall; Stroop Color and Word Test; Grooved Pegboard test; WRAT-3	Sleep fragmentation and poorer sleep quality have implications for neurocognitive functioning in obese adolescents
Xanthopoulos et al. (2014)	38 adolescents (mean age 14.3 years) with OSA and obsesity 21 obese adolescent controls 36 lean adolescent controls	Neuro-behavioral evaluation	Obese adolescents with OSA show impaired executive and behavioral function compared to obese and lean controls
Vitelli et al. (2015)	36 children with OSA 38 children with OSA and obesity	WISC-III	Obese children with OSA showed higher cognitive impairment
Lau et al. (2015)	23 OSA children (aged 8–12 years)	Working memory and basic attention tasks	Verbal working memory impairments are associated with OSA

*ABAS-II* adaptive behavior assessment system (Second Edition), *ADHD* attention deficit hyperactivity disorder, *BRIEF* behavior rating inventory of executive function, *CCT* children’s category test, *COWAT* controlled oral word association test, *CPT* continuous performance task, *DA* delayed alternation (The Shape School), *DAS* differential ability scales, *fMRI* functional magnetic resonance imaging, *NEPSY* Stanford-Binet intelligence scales for early childhood (Fifth Edition), neuropsychological assessment, *OSA* obstructive sleep apnea, *PS* primary snoring, *RAVLT* Rey auditory verbal learning test, *RBMT* Rivermead behavioral memory test for children, *RCFT* Rey complex figure test, *SR* Spatial reversal, *SWMT* sustained working memory task, *WASI* Wechsler abbreviated scale of intelligence, *WISC-III* Wechsler intelligence scale for children, *WIAT-II* Wechsler individual achievement test II, *WPPSI-R* Wechsler preschool and primary scale of intelligence, revised, *WRAT-3* wide range achievement test (3rd Edition)

In one of their studies, Jackman et al. (2012) focused on children (aged 3–5 years) with primary snoring and mild/moderate/severe OSA syndrome compared to non-snoring children. Overnight polysomnography and cognitive battery were applied, along with behavioral analyses. The authors reported that sleep-disordered breathing (SDB) of any severity was associated with poorer behavior, but not decreased cognitive performance. The authors also drew attention to the possible study limitations, such as the fact that during cognitive assessments of young children, it is often more difficult to keep them engaged in assessment procedures as they are also more easily distracted than older children. Also, children of this age may often refuse to complete subtests or are not sufficiently cooperative.

Jackman et al. (2012) also suggested that early treatment of OSA may prevent cognitive deficits arising later in childhood. Unfortunately, there is evidence that cognitive impairment is already found in children as young as 5 years. For example, Gottlieb et al. (2004) found that SDB symptoms are associated with poorer executive function and memory skills and lower general intelligence in children aged 5 years (using a parent-completed questionnaire, polysomnography, and standardized neurocognitive tests). Moreover, when school-aged children with SDB were treated with adenotonsillectomy, improvements in SDB after 4 years were concomitant with improvements in some areas of neurocognition, but not in academic ability or behavior (Biggs et al., 2014). Therefore, an earlier diagnosis (before age 5) and treatment may have prevented some of these cognitive deficits from occurring.

As mentioned above, there have been more studies on school-aged children. Bourke et al. (2011) found that neurocognitive deficits are common in children with SDB (aged 7–12 years) regardless of the disease severity. Archbold et al. (2004) examined 12 children with mild SBD (aged 8.0–11.9 years) and found they showed significant impairment in sustained attention and vigilance on a computerized continuous performance test. While a number of studies present cognitive domains that are similarly affected in both adults and children, such as executive functioning or attention, specific impaired domains for the pediatric population, such as phonological processing, have also been reported. For example, O’Brien et al. (2004) showed that children with SDB scored significantly lower than controls on the attention/executive function domain, and specifically on the visual attention and executive function. In addition, children with SDB scored significantly lower than controls on phonological processing, the skill that is critical for learning how to read. In a study performed a few years later by Hamasaki Uema et al. (2007), it was observed that children aged 6–12 years with OSA show worse Rey test scores, which could indicate deficits in a number of cognitive domains, such as visuo-spatial memory, attention, and working memory. In addition, Lau et al. (2015) found that verbal working memory impairment related to OSA in children may have a further negative impact on learning potential and neurocognitive development. However, it is important to note that Biggs et al. (2011) examined 127 children aged 7–12 years with SDB to find out whether the deficits in memory were due to parental overestimation, and found that parents of children with less severe SDB tend to overestimate their symptoms. This suggests that observation of deficits in working memory may be largely dependent on the assessment method, and that children with SDB may not be as impaired as previously thought.

Some unique cognitive deficits in school-aged children with OSA have been described compared with the adult population. For example, Kheirandish-Gozal et al. (2014) showed that cognitive and empathetic processing (as measured by a fMRI during a color-word Stroop task and an empathy task) was deteriorated in 10 children (aged 7–11 years) with polysomnographically diagnosed OSA. However, a much larger sample size is required to confirm this finding.

Another study in school-aged children found that children with OSA have a higher arousal index than controls (Miano et al. 2011). Therefore, they hypothesized that arousal is a defensive mechanism that may preserve cognitive function by counteracting the respiratory events at the expense of sleep maintenance. Moreover, they reported that children with OSA had a higher ADHD rating, which negatively correlated with the level of oxygen saturation during the night. This suggests a correlation between attention deficits and OSA in children, most likely due to hypoxia.

Similar to the findings in adults, neuroimaging or neuroelectrophysiological studies can confirm the cognitive deficits in children with OSA. For example, Quan et al. (2013) examined children with SDB by neurocognitive battery and electroencephalographic monitoring, and showed that long-term changes in executive function were undetectable by neuropscyhological testing, but were visible during neuroelectrophysiological monitoring. Moreover, in a study by Barnes et al. (2012), children with OSA underwent overnight sleep studies and neuropscyhological testing, as well as the oddball attention task, while event-related potentials (ERPs) were recorded. They concluded that specific ERPs during a single attention task can reliably identify the presence of OSA-associated cognitive dysfunction. According to authors, electrophysiological approaches during specific cognitive tasks may serve as simple, complementary, and reliable reporters of cognitive dysfunction associated with OSA in children.

The effects of obesity on cognitive deficits in children with OSA have also been investigated. In a study by Tan et al. (2014), 31 adolescents (aged 10–18 years) with OSA and obesity (mean BMI of 32.3 ± 4.9) were assessed according to intelligence, memory, learning, academic achievement, behavior, and executive functioning. The authors concluded that children with OSA and obesity may have an increased risk for some poorer educational and behavioral outcomes. In addition, Hannon et al. (2012) postulated that sleep fragmentation and poorer sleep quality have implications for neurocognitive functioning in obese adolescents. Similar conclusions were drawn by the research group from the Children’s Hospital of Philadelphia, who found that obese adolescents with OSA presented impaired executive and behavioral functions compared to controls, and were more likely to score in the clinically abnormal range on measures of neurobehavioral functioning (Xanthopoulos et al. 2014). Similarly, in the study performed by Vitelli et al. (2015), it was observed that in the group of children with OSA, the worst cognitive performance was found in the subgroup with comorbid obesity. According to the authors, these findings were very important, as this period of life is crucial for the development of proper frontal lobe functioning.

There are also other comorbidities described in the literature associated with OSA in children, such as Down syndrome (Lal et al. 2015). Among children with this condition, mean verbal IQ score was found to be lower in patients with comorbid OSA than in those without OSA, and performance on measures of cognitive flexibility was poorer (Breslin et al. 2014).

In summary, the cognitive dysfunctions in children focus on slightly different areas of cognition than in adults, such as behavioral and educational aspects, visual attention, and phonological processing, which are necessary for the proper intellectual development of a child. Based on the early changes in neuroimaging and neuropsychological presentation, these children should be treated early to prevent persistent, irreversible cognitive changes in the future. There should be more direct focus on assessing the cognitive functions in the youngest patients with OSA, however, due to their inability to verbally express their thoughts, there is limited ability for neuropsychological assessment.

### OSA treatment methods

#### OSA treatment in adults using continuous positive airway pressure

In terms of treatment in the adult population, the dominant method remains continuous positive airway pressure (CPAP), which requires strong patient compliance (Hussain et al. 2014). However, CPAP studies in OSA patients show a significant discrepancy in results, most likely related to a large variability in study designs and durations of treatment. Several authors (Aloia et al. 2004; Saunamäki and Jekhonen 2007; Lau et al. 2010; Antic et al. 2011) report that even the most optimally treated OSA patients may not experience a complete reversal in attention and executive functions, due to the permanent brain alterations in severe cases (Ferini-Strambi et al. 2003). It confirms the findings of the apnea positive pressure long-term efficacy study (APPLES), a recent, randomized, double-blind multi-center trial (Kushida et al. 2012) on 1098 participants diagnosed with OSA. APPLES reported that neurocognitive measures (attention and psychomotor function, verbal learning and memory, executive and frontal-lobe function) did not improve after 6 months of CPAP therapy compared to sham CPAP therapy. However, results of an earlier study (Felver-Gant et al. 2007) indicated that patients who were highly compliant with treatment demonstrated selective improvement in working memory after 3 months of CPAP treatment. Additionally, in a study on severe OSA patients (Ferini-Strambi et al. 2003) authors reported that after 15-days of CPAP treatment, attentive, visuo-spatial learning, and motor performances returned to normal baseline levels, but after a further 4 months of CPAP treatment did not result in any further improvement in cognitive tests. Moreover, they found that performance during tests evaluating executive functions and constructional abilities was not affected by short- and long-term treatment with CPAP.

One study showed that attention and vigilance, psychomotor speed, executive functioning, and non-verbal delayed recall improved in patients compliant with CPAP treatment after 3 months (Aloia et al. 2003). However, the results from studies investigating the effects of CPAP on memory are inconsistent. Grenèche et al. (2013) investigated the effects of CPAP treatment on short-term memory performance over 24 h of sustained wakefulness and revealed that untreated OSA patients had no deficit in the immediate memory but were impaired in working memory, which was improved after 6 months of CPAP treatment.

As the abnormalities in cognitive functions are reflected in structural brain changes, O’Donoghue et al. (2005) investigated the effect of CPAP treatment on brain structure. The authors reported no longitudinal changes in gray matter density or regional volumes after treatment, but whole brain volume decreased slightly. In contrast, Canessa et al. (2011) observed significant improvements involving memory, attention, and executive functioning that paralleled with gray-matter volume increases in hippocampal and frontal structures after 3 months of CPAP treatment. White matter integrity measured by diffusion tensor imaging (DTI) and cognitive performance was also found to improve in compliant patients after 12 months of CPAP treatment (Castronovo et al. 2014).

The promising results and return of some cognitive functions after compliant CPAP therapy indicate that more research in this area is warranted. Together with neuroimaging evaluation and high compliance to treatment, it would be possible to establish the proper duration, intensity and frequency of CPAP treatment in patients with cognitive dysfunctions.

#### OSA treatment in children using adenotonsillectomy and positive airway pressure

In the pediatric population, adenotonsillectomy is considered the most effective treatment (Lee et al. 2014). The most important clinical study on this treatment method, the CHAT clinical trial, was published in 2013 (Marcus et al. 2013). The CHAT study evaluated the usefulness of early adenotonsillectomy in a children aged 5–9 years in terms of a reduction in symptoms compared with a control group managed by watchful waiting and supportive care treatment. After evaluating 464 children, the authors concluded that surgical treatment for the OSA in school-age children did not significantly improve attention or executive function, as measured by neuropsychological testing, compared to watchful waiting management. However, adenotonsillectomy did reduce symptoms and improve secondary outcomes of behavior, quality of life, and polysomnographic findings. Similarly, Esteller et al. (2014) performed a study to analyze the outcomes of cognitive and behavioral disorders 1-year post-adenotonsillectomy. They found that behavioral and cognitive disturbances in children with OSA (aged 3–13 years) were partially resolved following adenotonsillectomy. However, improvements in the cognitive and behavioral variables did not significantly differ from the normal development of an individual, and were independent of the resolution of respiratory disorders.

Yuan et al. (2012) proposed that neurocognitive functioning in children with OSA may be improved with the use of positive airway pressure (PAP). Participants in their study were examined with the Wechsler Intelligence Scale for Children-IV, the Delis Kaplan Executive Functioning Scales, the Test of Everyday Attention for Children, and the Wide Range Assessment of Memory and Learning-2nd Edition to assess the neurocognitive function. Participants prior to therapy reflected neurocognitive deficiencies in all areas. Of the original 21 children, four completed the full PAP treatment and were re-evaluated, demonstrating improvements in memory and motor speed. While this study shows promising results, the sample size is too small to make any assumptions. Therefore, more research in the field of PAP treatment in children with neurocognitive dysfunctions due to OSA is required.

## Conclusions

Cognitive deficits related to breathing disorders have been observed in adults, children and adolescents, and show a wide range of neuropsychological presentations. In children, the symptoms may present as problems with working memory, attention, or executive functions cognitive domains, but also as changes in behavior and phonological processing necessary for proper development. However, if treated properly and early, these changes may be reversible (Idiazabal-Alecha and Fernandez-Prats 2014). Altered cognition accompanying breathing disorders has also been observed in adults (Varga et al. 2014) and elderly patients (Martin et al. 2014), and executive functions, attention and memory are the most affected cognitive domains in OSA (Bucks et al. 2013). These symptoms may be additionally complicated by depression and anxiety, which are more increasingly present in the adult population (Rezaeitalab et al. 2014). Despite much research, the prevalence and pathogenesis of breathing disorders in the different age groups remains unclear. Also, there is a need to set standardized protocols for the treatment of impaired cognitive functions associated with OSA. As highlighted in this review, the last decades of research in this field have resulted in inconsistent data, in part because of small sample sizes, different study designs, as well as varying test batteries, exclusion and inclusion criteria, and treatment durations. The literature devoted to cognition in OSA in children and adolescents is very limited and more studies should be performed on specific pediatric age groups. Finally, while PAP and adenotonsillectomy are currently the most effective treatments for OSA, further study is required to establish the most optimal treatment approach for each age group to prevent cognitive dysfunctions.

## References

[CR1] Alchanatis M, Deligiorgis N, Zias N, Amfilochiou A, Gotsis E, Karakatsani A, Papadimitriouz A (2004). Frontal brain lobe impairment in obstructive sleep apnoea: a proton MR spectroscopy study. Eur Respir J.

[CR2] Aloia MS, Ilniczky N, Di Dio P, Perlis ML, Greenblatt DW, Giles DE (2003). Neuropsychological changes and treatment compliance in older adults with sleep apnea. J Psychosom Res.

[CR3] Aloia MS, Arnedt JT, Davis JD, Riggs RL, Byrd D (2004). Neuropsychological sequelae of obstructive sleep apnea-hypopnea syndrome: a critical review. J Int Neuropsychol Soc.

[CR4] Antic NA, Catcheside P, Buchan C, Hensley M, Naughton MT, Rowland S, Williamson B, Windler S, McEvoy RD (2011). Sleep.

[CR5] Antonelli Incalzi R, Marra C, Salvigni BL, Petrone A, Gemma A, Selvaggio D, Mormile F (2004). Does cognitive dysfunction conform to a distinctive pattern in obstructive sleep apnea syndrome?. J Sleep Res.

[CR6] Archbold KH, Giordani B, Ruzicka DL, Chervin RD (2004). Cognitive executive dysfunction in children with mild sleep-disordered breathing. Biol Res Nurs.

[CR7] Atanasov AT, Dimov PD (2003). Nasal and sleep cycle—possible synchronization during night sleep. Med Hypotheses.

[CR8] Ayalon L, Ancoli-Israel S, Drummond SP (2009). Altered brain activation during response inhibition in obstructive sleep apnea. J Sleep Res.

[CR9] Barnes ME, Gozal D, Molfese DL (2012). Attention in children with obstructive sleep apnoea: an event-related potentials study. Sleep Med.

[CR10] Bartlett DJ, Rae C, Thompson CH, Byth K, Joffe DA, Enright T, Grunstein RR (2004). Hippocampal area metabolites relate to severity and cognitive function in obstructive sleep apnea. Sleep Med.

[CR11] Beebe DW, Gozal D (2002). Obstructive sleep apnea and the prefrontal cortex: towards a comprehensive model linking nocturnal upper airway obstruction to daytime cognitive and behavioral deficits. J Sleep Res.

[CR12] Beebe DW, Groesz BA, Wells C, Nichols A, McGee K (2003). The neuropsychological effects of obstructive sleep apnea: a meta-analysis of norm-referenced and case-controlled data. Sleep.

[CR13] Beebe DW, Wells CT, Jeffries J, Chini B, Kalra M, Amin R (2004). Neuropsychological effects of pediatric obstructive sleep apnea. J Int Neuropsychol Soc JINS.

[CR14] Biggs SN, Bourke R, Anderson V, Jackman AR, Killedar A, Nixon GM, Davey MJ, Walker AM, Trinder J, Horne RS (2011). Working memory in children with sleep-disordered breathing: objective versus subjective measures. Sleep Med.

[CR15] Biggs SN, Vlahandonis A, Anderson V, Bourke R, Nixon GM, Davey MJ, Horne RS (2014). Long-term changes in neurocognition and behavior following treatment of sleep disordered breathing in school-aged children. Sleep.

[CR16] Borges JG, Ginani GE, Hachul H, Cintra FD, Tufik S, Pompéia S (2013). Executive functioning in obstructive sleep apnea syndrome patients without comorbidities: focus on the fractionation of executive functions. J Clin Exp Neuropsychol.

[CR17] Borges PD, da Silva BB, Moita Neto JM, Borges NE, Li LM (2014). Cephalometric and anthropometric data of obstructive apnea in different age groups. Braz J Otorhinolaryngol.

[CR18] Bourke R, Anderson V, Yang JS, Jackman AR, Killedar A, Nixon GM, Davey MJ, Walker AM, Trinder J, Horne RS (2011). Cognitive and academic functions are impaired in children with all severities of sleep-disordered breathing. Sleep Med.

[CR19] Breslin J, Spano G, Bootzin R, Anand P, Nadel L, Edgin J (2014). Obstructive sleep apnea syndrome and cognition in Down syndrome. Dev Med Child Neurol.

[CR20] Bucks RS, Olaithe M, Eastwood P (2013). Neurocognitive function in obstructive sleep apnoea: a meta-review. Respirology.

[CR21] Canessa N, Castronovo V, Cappa SF, Aloia MS, Marelli S, Falini A, Alemanno F, Ferini-Strambi L (2011). Obstructive sleep apnea: brain structural changes and neurocognitive function before and after treatment. Am J Respir Crit Care Med.

[CR22] Castronovo V, Scifo P, Castellano A, Aloia MS, Iadanza A, Marelli S, Cappa SF, Strambi LF, Falini A (2014) White matter integrity in obstructive sleep apnea before and after treatment. Sleep 37:sp-00071-13 10.5665/sleep.3994 PMC4153061 25142557

[CR23] Chen X, Pensuksan WC, Lohsoonthorn V, Lertmaharit S, Gelaye B, Williams M (2014). Obstructive sleep apnea and multiple anthropometric indices of general obesity and abdominal obesity among young adults. Int J Soc Sci Stud.

[CR001] Djonlagic I, Guo M, Matteis P, Carusona A, Stickgold R, Malhotra A (2014). Untreated sleep-disordered breathing: links to aging-related decline in sleep-dependent memory consolidation. PLoS One.

[CR24] Esteller E, Barceló M, Segarra F, Estivill E, Girabent-Farrés M (2014). Neurocognitive and behavioral disturbances after adenotonsillectomy in obstructive sleep apnea syndrome. An Pediatr (Barc).

[CR25] Felver-Gant JC, Bruce AS, Zimmerman M, Sweet LH, Millman RP, Aloia MS (2007). Working memory in obstructive sleep apnea: construct validity and treatment effects. J Clin Sleep Med.

[CR26] Ferini-Strambi L, Baietto C, Di Gioia MR, Castaldi P, Castronovo C, Zucconi M, Cappa SF (2003). Cognitive dysfunction in patients with obstructive sleep apnea (OSA): partial reversibility after continuous positive airway pressure (CPAP). Brain Res Bull.

[CR27] Findley LJ, Barth JT, Powers DC, Wilhoit SC, Boyd DG, Suratt PM (1986). Cognitive impairment in patients with obstructive sleep apnea and associated hypoxemia. Chest.

[CR28] Gagnon K, Baril AA, Gagnon JF, Fortin M, Décary A, Lafond C, Desautels A, Montplaisir J, Gosselin N (2014). Cognitive impairment in obstructive sleep apnea. Pathol Biol (Paris).

[CR29] Gelir E, Basaran C, Bayrak S, Yagcioglu S, Budak MT, Firat H, Ungan P (2014). Electrophysiological Assessment of the effects of obstructive sleep apnea on cognition. PLoS One.

[CR30] Gottlieb DJ, Chase C, Vezina RM, Heeren TC, Corwin MJ, Auerbach SH, Weese-Mayer DE, Lesko SM (2004). Sleep-disordered breathing symptoms are associated with poorer cognitive function in 5-year-old children. J Pediatr.

[CR31] Greenberg GD, Watson RK, Deptula D (1987). Neuropsychological dysfunction in sleep apnea. Sleep.

[CR32] Grenèche J, Krieger J, Bertrand F, Erhardt C, Maumy M, Tassi P (2013). Effect of continuous positive airway pressure treatment on short-term memory performance over 24 h of sustained wakefulness in patients with obstructive sleep apnea-hypopnea syndrome. Sleep Med.

[CR33] Guilleminault C, Eldridge FL, Tilkian A, Simmons FB, Dement WC (1977). Sleep apnea syndrome due to upper airway obstruction: a review of 25 cases. Arch Intern Med.

[CR34] Hamasaki Uema SF, Nagata Pignatari SS, Fujita RR, Moreira GA, Pradella-Hallinan M, Weckx L (2007). Assessment of cognitive learning function in children with obstructive sleep breathing disorders. Braz J Otorhinolaryngol.

[CR35] Hannon TS, Rofey DL, Ryan CM, Clapper DA, Chakravorty S, Arslanian SA (2012). Relationships among obstructive sleep apnea, anthropometric measures, and neurocognitive functioning in adolescents with severe obesity. J Pediatr.

[CR36] Health Quality Ontario (2006). Polysomnography in patients with obstructive sleep apnea: an evidence-based analysis. Ont Health Technol Assess Ser.

[CR37] Hilsendager CA, Zhang D, McRae C, Aloia M (2015). Assessing the influence of obesity on longitudinal executive functioning performance in patients with obstructive sleep apnea syndrome. Obes Res Clin Pract.

[CR38] Horiuchi F, Oka Y, Komori K, Tokui Y, Matsumoto T, Kawabe K, Ueno S (2014). Effects of adenotonsillectomy on neurocognitive function in pediatric obstructive sleep apnea syndrome. Case Rep Psychiatry..

[CR39] Hussain SF, Irfan M, Waheed Z, Alam N, Mansoor S, Islam M (2014). Compliance with continuous positive airway pressure (CPAP) therapy for obstructive sleep apnea among privately paying patients—a cross sectional study. BMC Pulm Med.

[CR40] Idiazabal-Alecha MA, Fernandez-Prats M (2014). Sleep-disordered breathing in early childhood: their neurocognitive repercussions. Revista de Neurol.

[CR41] Jackman AR, Biggs SN, Walter LM, Embuldeniya US, Davey MJ, Nixon GM, Anderson V, Trinder J, Horne RS (2012). Sleep-disordered breathing in preschool children is associated with behavioral, but not cognitive, impairments. Sleep Med.

[CR42] Joo EY, Jeon S, Kim ST, Lee JM, Hong SB (2013). Localized cortical thinning in patients with obstructive sleep apnea syndrome. Sleep.

[CR43] Jurádo-Gámez B, Guglielmi O, Gude F, Buela-Casal G (2015). Effects of continuous positive airway pressure treatment on cognitive functions in patients with severe obstructive sleep apnoea. Neurologia.

[CR44] Kheirandish-Gozal L, Yoder K, Kulkarni R, Gozal D, Decety J (2014). Preliminary functional MRI neural correlates of executive functioning and empathy in children with obstructive sleep apnea. Sleep.

[CR45] Klonoff H, Fleetham J, Taylor R, Clark C (1987). Treatment outcome of obstructive sleep apnea: physiological and neuropsychological con- comitants. J Nerv Men Dis.

[CR002] Kumar R, Farahvar S, Ogren JA, Macey PM, Thompson PM, Woo MA, Yang-Go FL, Harper RM (2014). Brain putamen volume changes in newly-diagnosed patients with obstructive sleep apnea. Neuroimage Clin.

[CR46] Kushida CA, Nichols DA, Holmes TH, Quan SF, Walsh JK, Gottlieb DJ, Simon RD, Guilleminault C, White DP, Goodwin JL, Schweitzer PK, Leary EB, Hyde PR, Hirshkowitz M, Green S, McEvoy LK, Chan C, Gevins A, Kay GG, Bloch DA, Crabtree T, Demen WC (2012). Effects of continuous positive airway pressure on neurocognitive function in obstructive sleep apnea patients: the apnea positive pressure long-term efficacy study (APPLES). Sleep.

[CR47] Lal C, White DR, Joseph JE, van Bakergem K, LaRosa A (2015). Sleep-disordered breathing in Down syndrome. Chest.

[CR48] Lau EY, Eskes GA, Morrison DL, Rajda M, Spurr KF (2010). Executive function in patients with obstructive sleep apnea treated with continuous positive airway pressure. J Int Neuropsychol Soc.

[CR49] Lau EY, Choi EW, Lai ES, Lau KN, Au CT, Yung WH, Li AM (2015). Working memory impairment and its associated sleep-related respiratory parameters in children with obstructive sleep apnea. Sleep Med.

[CR50] Lee LA, Li HY, Lin YS, Fang TJ, Huang YS, Hsu JF, Wu CM, Huang CG (2014). Severity of childhood obstructive sleep apnea and hypertension improved after adenotonsillectomy. Otolaryngol Head Neck Surg Off J Am Acad Otolaryngol Head Neck Surg.

[CR51] Lis S, Krieger S, Hennig D, Röder C, Kirsch P, Seeger W, Gallhofer B, Schulz R (2008). Executive functions and cognitive subprocesses in patients with obstructive sleep apnoea. J Sleep Res.

[CR52] Liu H, Yuan X (2014). The difference and similarity of obesity hypoventilation syndrome and obstructive sleep apnea hypopnea syndrome. Chin J Tuberc Respir Dis.

[CR53] Macey PM, Kumar R, Woo MA, Valladares EM, Yan-Go FL, Harper RM (2008). Brain structural changes in obstructive sleep apnea. Sleep.

[CR54] Marcus CL, Moore RH, Rosen CL, Giordani B, Garetz SL, Taylor HG, Mitchell RB, Amin R, Katz ES, Arens R, Paruthi S, Muzumdar H, Gozal D, Thomas NH, Ware J, Beebe D, Snyder K, Elden L, Sprecher RC, Willging P, Jones D, Bent JP, Hoban T, Chervin RD, Ellenberg SS, Redline S, Childhood Adenotonsillectomy Trial (CHAT) (2013). A randomized trial of adenotonsillectomy for childhood sleep apnea. N Engl J Med.

[CR55] Martin MS, Sforza E, Roche F, Barthelemy JC, Thomas-Anterion C (2014) Sleep breathing disorders and cognitive function in the elderly: an 8-year follow-up study. The proof-synapse cohort. Sleep 10.5665/sleep.4392 PMC4288598 25325480

[CR56] Mazza S, Pépin JL, Naëgelé B, Plante J, Deschaux C, Lévy P (2005). Most obstructive sleep apnoea patients exhibit vigilance and attention deficits on an extended battery of tests. Eur Respir J.

[CR57] Miano S, Paolino MC, Urbano A, Parisi P, Massolo AC, Castaldo R, Villa MP (2011). Neurocognitive assessment and sleep analysis in children with sleep-disordered breathing. Clin Neurophysiol Off J Int Fed Clin Neurophysiol.

[CR58] Morrell MJ, McRobbie DW, Quest RA, Cummin AR, Ghiassi R, Corfield DR (2003). Changes in brain morphology associated with obstructive sleep apnea. Sleep Med.

[CR59] Naëgelé B, Launois SH, Mazza S, Feuerstein C, Pépin JL, Lévy P (2006). Which memory processes are affected in patients with obstructive sleep apnea? An evaluation of 3 types of memory. Sleep.

[CR60] Nemeth D, Csábi E, Janacsek K, Várszegi M, Mari Z (2012). Intact implicit probabilistic sequence learning in obstructive sleep apnea. J Sleep Res.

[CR61] O’Brien LM, Mervis CB, Holbrook CR, Bruner JL, Smith NH, McNally N, McClimment MC, Gozal D (2004). Neurobehavioral correlates of sleep-disordered breathing in children. J Sleep Res.

[CR62] O’Donoghue FJ, Briellmann RS, Rochford PD, Abbott DF, Pell GS, Chan CH, Tarquinio N, Jackson GD, Pierce RJ (2005). Cerebral structural changes in severe obstructive sleep apnea. Am J Respir Crit Care Med.

[CR63] Paiva T, Attarian H (2014). Obstructive sleep apnea and other sleep-related syndromes. Handb Clin Neurol.

[CR64] Pierobon A, Giardini A, Fanfulla F, Callegari S, Majani G (2008). A multidimensional assessment of obese patients with obstructive sleep apnoea syndrome (OSAS): a study of psychological, neuropsychological and clinical relationships in a disabling multifaceted disease. Sleep Med.

[CR65] Quan SF, Wright R, Baldwin CM, Kaemingk KL, Goodwin JL, Kuo TF, Kaszniak A, Boland LL, Caccappolo E, Bootzin RR (2006). Obstructive sleep apnea-hypopnea and neurocognitive functioning in the Sleep Heart Health Study. Sleep Med.

[CR66] Quan SF, Archbold K, Gevins AS, Goodwin JL (2013). Long-term neurophysiologic impact of childhood sleep disordered breathing on neurocognitive performance. Southwest J Pulm Crit Care.

[CR67] Rezaeitalab F, Moharrari F, Saberi S, Asadpour H, Rezaeetalab F (2014). The correlation of anxiety and depression with obstructive sleep apnea syndrome. J Res Med Sci Off J Isfahan Univ Med Sci.

[CR68] Rouleau I, Décary A, Chicoine AJ, Montplaisir J (2002). Procedural skill learning in obstructive sleep apnea syndrome. Sleep.

[CR69] Ryan S, Crinion SJ, McNicholas WT (2014). Obesity and sleep-disordered breathing- when two ‘bad guys’ meet. QJM Mon J Assoc Physicians.

[CR003] Sales LV, Bruin VM, D’Almeida V, Pompeia S, Bueno OF, Tufik S, Bittencourt L (2013). Cognition and biomarkers of oxidative stress in obstructive sleep apnea. Clinics (Sao Paulo).

[CR70] Saunamäki T, Jehkonen M (2007). A review of executive functions in obstructive sleep apnea syndrome. Acta Neurol Scand.

[CR71] Saunamäki T, Himanen SL, Polo O, Jehkonen M (2009). Executive dysfunction in patients with obstructive sleep apnea syndrome. Eur Neurol.

[CR72] Sforza E, Haba-Rubio J, De Bilbao F, Rochat T, Ibanez V (2004). Performance vigilance task and sleepiness in patients with sleep-disordered breathing. Eur Respir J.

[CR73] Solyom R, Csiszer I, Neagos A (2014). Tonsillar hypertrophy implications in sleep disorders in adults and children. Rom J Morphol Embryol.

[CR74] Tan E, Healey D, Schaughency E, Dawes P, Galland B (2014). Neurobehavioural correlates inolder children and adolescents with obesity and obstructive sleep apnoea. J Paediatr Child Health.

[CR75] Thomas RJ, Rosen BR, Stern CE, Weiss JW, Kwong KK (2005). Functional imaging of working memory in obstructive sleep-disordered breathing. J Appl Physiol.

[CR76] Torelli F, Moscufo N, Garreffa G, Placidi F, Romigi A, Zannino S, Bozzali M, Fasano F, Giulietti G, Djonlagic I, Malhotra A, Marciani MG, Guttmann CRG (2011). Cognitive profile and brain morphological changes in obstructive sleep apnea. NeuroImage.

[CR77] Tulek B, Atalay NB, Kanat F, Suerdem M (2013). Attentional control is partially impaired in obstructive sleep apnea syndrome. J Sleep Res.

[CR78] Twigg GL, Papaioannou I, Jackson M, Ghiassi R, Shaikh Z, Jaye J, Graham KS, Simonds AK, Morrell MJ (2010). Obstructive sleep apnea syndrome is associated with deficits in verbal but not visual memory. Am J Respir Crit Care.

[CR79] Varga AW, Kishi A, Mantua J, Lim J, Koushyk V, Leibert DP, Osorio RS, Rapoport DM, Ayappa I (2014). Apnea-induced rapid eye movement sleep disruption impairs human spatial navigational memory. J Neurosci Off J Soc Neurosci.

[CR004] Verstraeten E, Cluydts R, Pevernagie D, Hoffman G (2004). Executive function in sleep apnea: controlling for attentional capacity in assessing executive attention. Sleep.

[CR80] Vitelli O, Tabarrini A, Miano S, Rabasco J, Pietropaoli N, Forlani M, Parisi P, Villa MP (2015). Impact of obesity on cognitive outcome in children with sleep-disordered breathing. Sleep Med.

[CR81] Wallace A, Bucks RS (2013). Memory and obstructive sleep apnea: a meta-analysis. Sleep.

[CR82] Xanthopoulos MS, Gallagher PR, Berkowitz RI, Radcliffe J, Bradford R, Marcus CL (2014) Neurobehavioral functioning in adolescents with and without obesity and obstructive sleep apnea. Sleep. pii: sp-00197-14 10.5665/sleep.4498 PMC4335517 25325469

[CR83] Yaouhi K, Bertran F, Clochon P, Mézenge F, Denise P, Foret J, Eustache F, Desgranges B (2009). A combined neuropsychological and brain imaging study of obstructive sleep apnea. J Sleep Res.

[CR84] Yuan HC, Sohn EY, Abouezzeddine T, Mahrer NE, Barber BA, Keens TG, Davidson Ward SL, Gold JI (2012). Neurocognitive functioning in children with obstructive sleep apnea syndrome: a pilot study of positive airway pressure therapy. J Pediatr Nurs.

[CR85] Zawada K, Burzynska-Kozmin A, Krysta K (2013). Patients with obesity hypoventilation syndrome and mental disorders. Eur Psychiatry.

[CR86] Zhang X, Ma L, Li S, Wang Y, Wang L (2011). A functional MRI evaluation of frontal dysfunction in patients with severe obstructive sleep apnea. Sleep Med.

